# In situ activation graphitization to fabricate hierarchical porous graphitic carbon for supercapacitor

**DOI:** 10.1038/s41598-021-85661-0

**Published:** 2021-03-25

**Authors:** Yanling Zhao, Xiaohua Zhang

**Affiliations:** 1 College of Medical Imaging, Shanxi Medical University, Taiyuan, 030001 China; 2 College of Materials Science and Engineering, Taiyuan University of Science and Technology, Taiyuan, 030024 China

**Keywords:** Carbon capture and storage, Porous materials

## Abstract

In situ activation–graphitization method based on the atomically dispersed K and Fe in organic salts is developed to synthesize hierarchical porous graphitic carbon by directly pyrolysis potassium citrate and iron citrate. Moreover, (NH_4_)_2_C_2_O_4_ is also employed as both N dopant and porogen to open up internal structure and regulate pore structure. The inside-out activation leads to the homogeneous reaction and interconnected hierarchical porous structure with few dead pores. Accompanied by high specific surface area, appropriate pore distribution, good conductivity, and N/O functional groups, the sample exhibits high capacitance of 322.6 F g^−1^ at 0.5 A g^−1^, good rate capability, and excellent cycling stability with 101.5% capacitance retention after 15,000 cycles. The supercapacitor shows an energy density of 21.3 W h kg^−1^ at 456.7 W kg^−1^ in 1 M Na_2_SO_4_. Easy synthesis, cost-effective, and environmentally benign, the work provides a promising strategy to produce hierarchical porous graphitic carbon applied in energy storage.

## Introduction

Energy storage devices play a pivotal role in the development of clean and sustainable energy, and they have been widely applied in electric vehicles, solar energy, wind power industry and so on^1,2^. Supercapacitors represent one of the promising energy storage technologies owing to their high power density, fast charge–discharge rate, and long cyclic stability, which are interesting for various clean-energy device systems^3,4^. Porous carbon (PC) materials offer numerous advantages for energy storage and show excellent electrochemical performances in supercapacitors, based on their large specific surface area (*S*_BET_), hierarchical porous structure, relatively good conductivity, and excellent chemical and mechanical stability^5,6^. Specifically, large *S*_BET_ can provide numerous active sides for charge storage; hierarchical porous structure can simultaneously serve as ion-buffering reservoirs (macropores), act as rapid ion transfer channels (mesopores), and provide abundant locations for ions accumulation (micropores); good conductivity can greatly enhance electron transport kinetics^7^. However, pore structure and electrical conductivity of carbon materials have a competitive relationship. A highly porous structure usually lead to poor electrical conductivity owing to excessive macropores and mesopores^8^. In contrast, good electrical conductivity is usually linked with a high graphitization degree, giving rise to undeveloped pore structure and a small *S*_BET_, which are unfavorable for ions transport and charge accommodation. On account of this, a rational balanced relationship between pore structure and electrical conductivity is of great significance to maximize the electrochemical performances.

Porous carbon, especially activated carbon, is often prepared by pyrolyzing carbon-rich organic materials with activating agents to develop hierarchical porosity and enhance *S*_BET_^9^. However, the graphitic structure is not desirable after chemical activation. Catalytic graphitization with transition metals as catalyst is an effective way to promote the formation of graphitic structure. Previous researches indicate that the combination of chemical activation and catalytic graphitization is an effective way to simultaneously improve pore structure and graphitization degree. For example, carbon precursors are treated with two different catalysts, which are used for chemical activation (pore-forming reagents: KOH, ZnCl_2_, or K_2_CO_3_) and catalytic graphitization (metals salts containing Fe, Co, or Ni)^8^. Chang et al. fabricated hierarchical porous carbon by metal ion pretreatment and a two-step post-activation^10^. The presence of catalyst Co(NO_3_)_2_ is able to balance the reduced graphitization caused by activation. Many porous graphitic carbon (PGC) materials can also be synthesized by one-step carbonization with FeCl_3_ and ZnCl_2_ as graphitization catalyst and chemical activator, respectively^11,12^. Although more effective, the majority of activating agents, such as KOH, ZnCl_2_, and H_3_PO_4_, are highly corrosive and harmful, which cause serious corrosion of equipment and environmental problems. To this end, it is highly desirable to develop facile and eco-friendly strategy to develop hierarchical porous graphitic carbon (HPGC) with hierarchical porous structure and high graphitization degree.

Recently, carbon materials derived from organic salts (such as potassium citrate, ferric citrate, calcium gluconate) have drawn considerable attention, owing to their merits of low-cost, well-defined molecular, and facile procedures for carbonization and graphitization^13–15^. Besides, organic salts have atomically dispersed metal species, such as K, Na, Ca, Fe, and so on. These dispersed metal species can be used as self-templates, activating agents, or graphitization catalysts. For example, the polymer containing K can be decomposed and produces K_2_CO_3_ during carbonization, creating pores in carbon framework^16^. As for iron species, it can be reduced by carbon to form Fe_3_C at high temperature, which is an important intermediate product for the formation of graphitic carbon^17^. In previous reports, potassium citrate and iron citrate have been respectively studied as carbon source to produce carbon material for applied in supercapacitors^18,19^. However, they only focused on the influences of porous structure, specific surface area, or electron conductivity on the electrochemical performances of carbon materials, without considering the competitive relationship between pore structure and electrical conductivity. In this paper, both potassium citrate and ferric citrate were used as carbon sources. Taking advantages of these two different organic salts, in situ activation−graphitization can be achieved. The obtained materials can balance the relationship between pore structure and graphitization degree. Moreover, we employed (NH_4_)_2_C_2_O_4_ as pore-forming agent and nitrogen precursor to increase the active surface area and provide additional pseudocapacitance^20^.

## Results

### Morphology and structural properties

**Figure 1:**
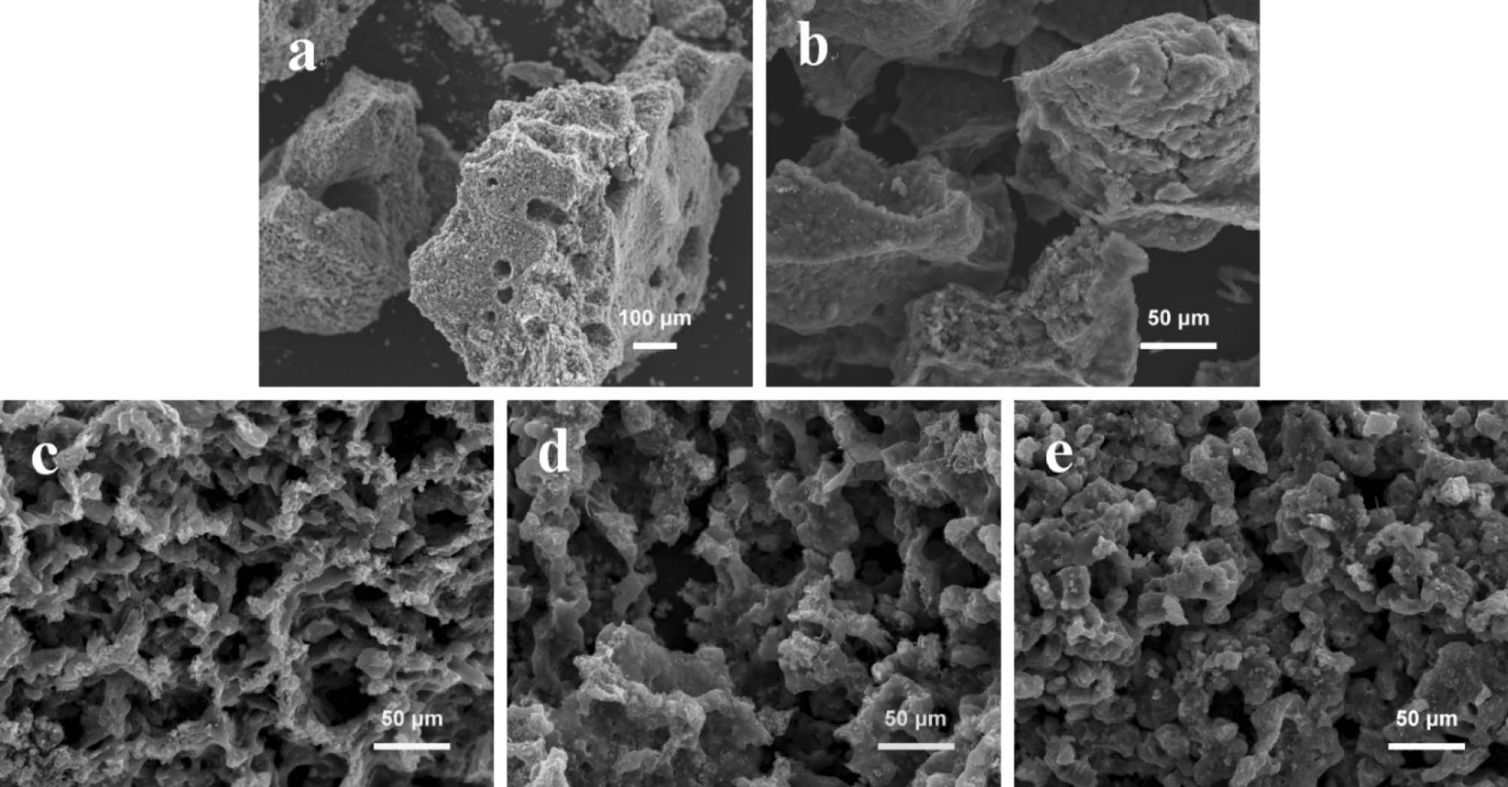
SEM images of (**a**) PC-750, (**b**) PGC-750, (**c**) HPGC-700, (**d**) HPGC-750, and (**e**) HPGC-800. Photographs compiled using Powerpoint 2019 (https://www.microsoft.com/zh-cn/microsoft-365/get-started-with-office-2019) without changing the content of images themselves.

**Figure 2:**
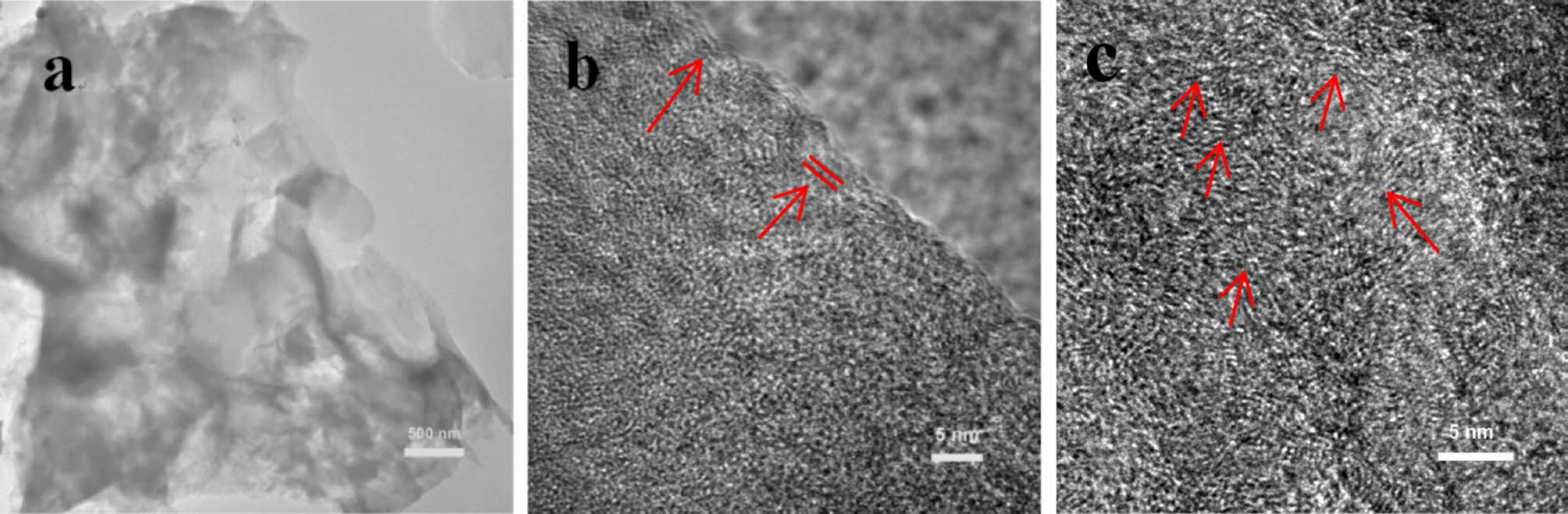
(**a**,**b**) TEM images of HPGC-750 under different magnifications; (**c**) TEM image of HPGC-800. Photographs compiled using Powerpoint 2019 (https://www.microsoft.com/zh-cn/microsoft-365/get-started-with-office-2019) without changing the content of images themselves.

During the high temperature treatment of potassium citrate, a certain amount of K compounds can be generated, such as K_2_CO_3_ and K_2_O. K_2_CO_3_ acts as a crucial role in the development of micropores, which can play as an activating agent and corrode carbon skeleton (K_2_CO_3_ + 2C → 2 K + 3CO). And the K vapors can intercalate into carbon materials, making swelling and disruption of carbon microstructure and creating additional porosity^18^. Besides, Fe_3_C can be formed during the thermal decomposition process of iron citrate, and it can transform amorphous carbon into graphitized carbon at high temperature. The morphology and pore geometry of the prepared carbon materials were observed by SEM images. Both PC-750 (Fig. 1a) and PGC-750 (Fig. 1b) exhibit a bulky monolithic morphology, and large pores are hard to be observed. The solid block of carbon with few macropores or mesopores is disadvantageous for ions transport and energy storage. Surprisingly, when (NH_4_)_2_C_2_O_4_ is incorporated during the carbonization process, the bulk carbon particles are converted into unique, well-defined 3D honeycomb framework constructed by hierarchical porous carbon skeletons (Fig. 1d), showing good pore connectivity. The macropores are evenly distributed in HPGC-750, which is attributed to physical activation effect of the chemically released gases (NH_3_, CO, CO_2_) from (NH_4_)_2_C_2_O_4_ during thermal pyrolysis. The different structures of PGC-750 and HPGC-750 indicate that the employment of (NH_4_)_2_C_2_O_4_ is contributed to the formation of interconnected porous structure. Figure 1c–e manifest that carbonization temperature is the key element influence the morphologies of carbon materials*.* With the increase of temperature, the honeycomb structure of HPGC-*T* step-by-step becomes irregular, which caused by the collapse of the carbon skeleton at high temperature^21^. TEM images also confirm that HPGC-750 has a honeycomb-like porous structure with macropores, mesopores (Fig. 2a) and dense micropores (Fig. 2b). The combination of macropores, mesopores, and micropores can buffer electrolyte to shorten ion diffusion distance and facilitate fast ion transport to the interior surface, providing numerous effective electrochemical sites. In Fig. 2b, some ordered lattic fringes with the interlayers spacing of ~ 0.34 nm, originated from the graphitization of carbon. More ordered lattic fringes can be observed in HPGC-800 (Fig. 2c), indicating a higher content of graphitic carbon.

**Figure 3:**
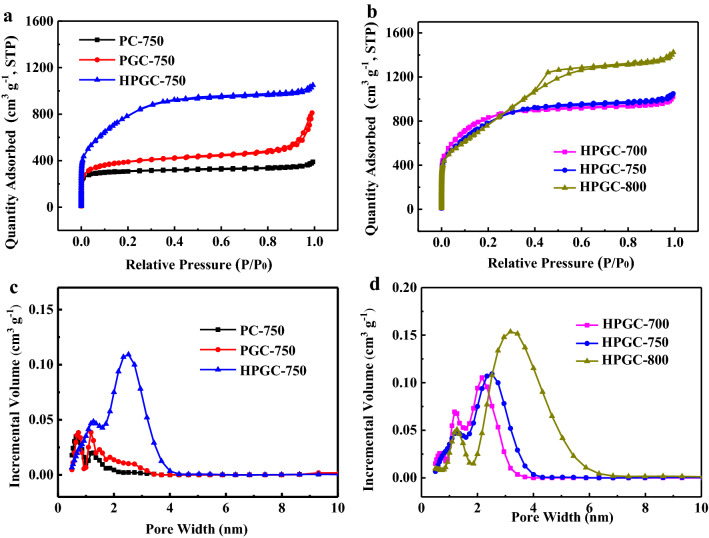
(**a**) N_2_ adsorption–desorption isotherms and (**c**) the pore size distribution curves of PC-750, PGC-750, and HPGC-750; (**b**) N_2_ adsorption–desorption isotherms and (**d**) the pore size distribution curves of HPGC-*T*.

The N_2_ adsorption–desorption isotherm of PC-750 in Fig. 3a displays a type-I isotherm, indicating the existence of a large amount of micropores, which originate from the in-suit activation effect of potassium citrate. PGC-750 shows a type-II isotherm with an increased adsorption at high relative pressure region (*P*/*P*_0_ > 0.9), revealing that PGC-750 consists of micropores and macropores. Obviously, when using (NH_4_)_2_C_2_O_4_ as pore-forming agent, the adsorbed quantity of HPGC-750 dramatically increases and the isotherm can be classified as type-IV isotherm, which is attributed to a large number of mesopores in the sample as verified by pore size distribution curves in Fig. 3c. Mesopore can effectively minimize ion transport resistance and improve the wettability of the material^22^. Detailed texture properties of the samples are summarized in Table 1. When only potassium citrate used, PC-750 has the lowest *S*_BET_ of 1194.0 m^2^ g^−1^ and the smallest total pore volume (*V*_total_, 0.60 cm^3^ g^−1^). With the presence of ferric citrate, *S*_BET_ and *V*_total_ of PGC-750 increase to 1445.0 cm^3^ g^−1^ and 1.25 cm^3^ g^−1^, respectively. The distinct differences in porosity properties suggest that the presence of Fe species offer additional templates to create pores and increase *S*_BET_. Significant increase in both *S*_BET_ and *V*_total_ have been observed in HPGC-750 because the released gases (NH_3_, CO, CO_2_, and H_2_O) from (NH_4_)_2_C_2_O_4_, which may act as pore-forming agents to create more pores^23^. Carbonization temperature also has a great influence on porous structure (i.e. *S*_BET_, pore size distribution, and total pore volume). As seen in Fig. 3b, HPGC-700 and HPGC-750 have the same features about isotherms, but the isotherm of HPGC-800 has obvious change. The *S*_BET_ of HPGC-*T* follows the trend of HPGC-700 (3118.3 m^2^ g^−1^) > HPGC-750 (2973.3 m^2^ g^−1^) > HPGC-800 (2951.1 m^2^ g^−1^). The pore size distribution curves in Fig. 3d indicate an enlargement of pore size, especially mesoporous, as the temperature increases. The average pore size enlarges from 2.03 nm (HPGC-700) to 2.99 nm (HPGC-800). These phenomenons are caused by the collapse of carbon skeleton at higher carbonization temperature.

**Table 1:** Porosity properties of as-prepared carbon samples.

Samples	*S*_BET_ (m^2^ g^−1^)	*S*_mic_^a^ (m^2^ g^−1^)	*S*_mes_^b^ (m^2^ g^−1^)	*V*_mic_^c^ (cm^3^ g^−1^)	*V*_total_^d^ (cm^3^ g^−1^)	*D*_ap_^e^ (nm)
PC-750	1194.0	573.9	620.1	0.49	0.60	2.02
PGC-750	1445.0	574.0	871	0.53	1.25	3.47
HPGC-700	3118.3	1589.1	1529.2	1.39	1.58	2.03
HPGC-750	2973.3	1586.4	1386.9	1.45	1.62	2.18
HPGC-800	2951.1	1597.2	1353.9	1.97	2.21	2.99

^a^Micropore surface area.

^b^Mesopore surface area.

^c^Micropore volume.

^d^Total pore volume.

^e^Average pore size.

**Figure 4:**
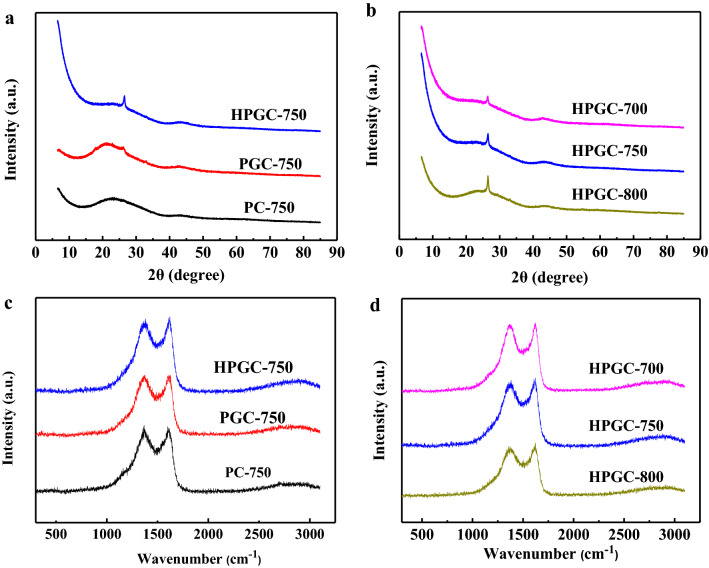
(**a**) XRD patterns and (**c**) Raman spectra of PC-750, PGC-750, and HPGC-750; (**b**) XRD patterns and (**d**) Raman spectra of HPGC-*T*.

The graphitic structures of the prepared carbon materials were investigated by XRD and Raman. As shown in Fig. 4a, both PC-750 and PGC-750 show a broad diffraction peak (2*θ* ≈ 20–30°), which is the characteristic of amorphous carbon framework. While, PGC-750 has an additional peak at 2*θ* = 26.26° assigned to the (002) diffraction of graphitic carbon, indicating its partly graphitic structure. Interestingly, with the presence of (NH_4_)_2_C_2_O_4_, the intensity of (002) diffraction peak of HPGC-750 becomes sharper and stronger, suggesting a higher graphitization degree. Moreover, a higher intensity of peak at the low-angle scattering region indicates a high density of micropores, thus providing more active sites for charge storage. Carbonization temperature is one of the key factors affecting the graphitic structure of carbon materials. As shown in Fig. 4b, high temperature results in an increased intensity of (002) peak, which is related to the development of amorphous structure towards the graphitic structure.

Raman spectra further prove the characteristic of disorder carbon and graphitic carbon (Fig. 4c,d). All Raman spectra of samples show the well-known D-band (stemming from the disordered carbon) at around 1375 cm^−1^ and typical G-bond (originating from the sp^2^-hybridized graphitic carbon) at around 1620 cm^−1^. The *I*_G_/*I*_D_ value of carbon material can be taken as an index to reflect the graphitic degree. The *I*_G_/*I*_D_ values of PC-750, PGC-750, and HPGC-750 are 0.99, 1.00, and 1.02, respectively, which are significantly higher than that of commercial Norit activated carbon (*I*_G_/*I*_D_ = 0.52)^24^. In addition, the *I*_G_/*I*_D_ value increases from 1.00 (HPGC-700) to 1.02 (HPGC-800) with carbonization temperature increases from 700 to 800 °C, which is in accordance with the XRD patterns. The appropriate coexistence of graphitic and amorphous carbon could enhance electrical conductivity, improve wettability, and increase active surface area, benefitting for capacitance improvement^22^.

**Figure 5:**
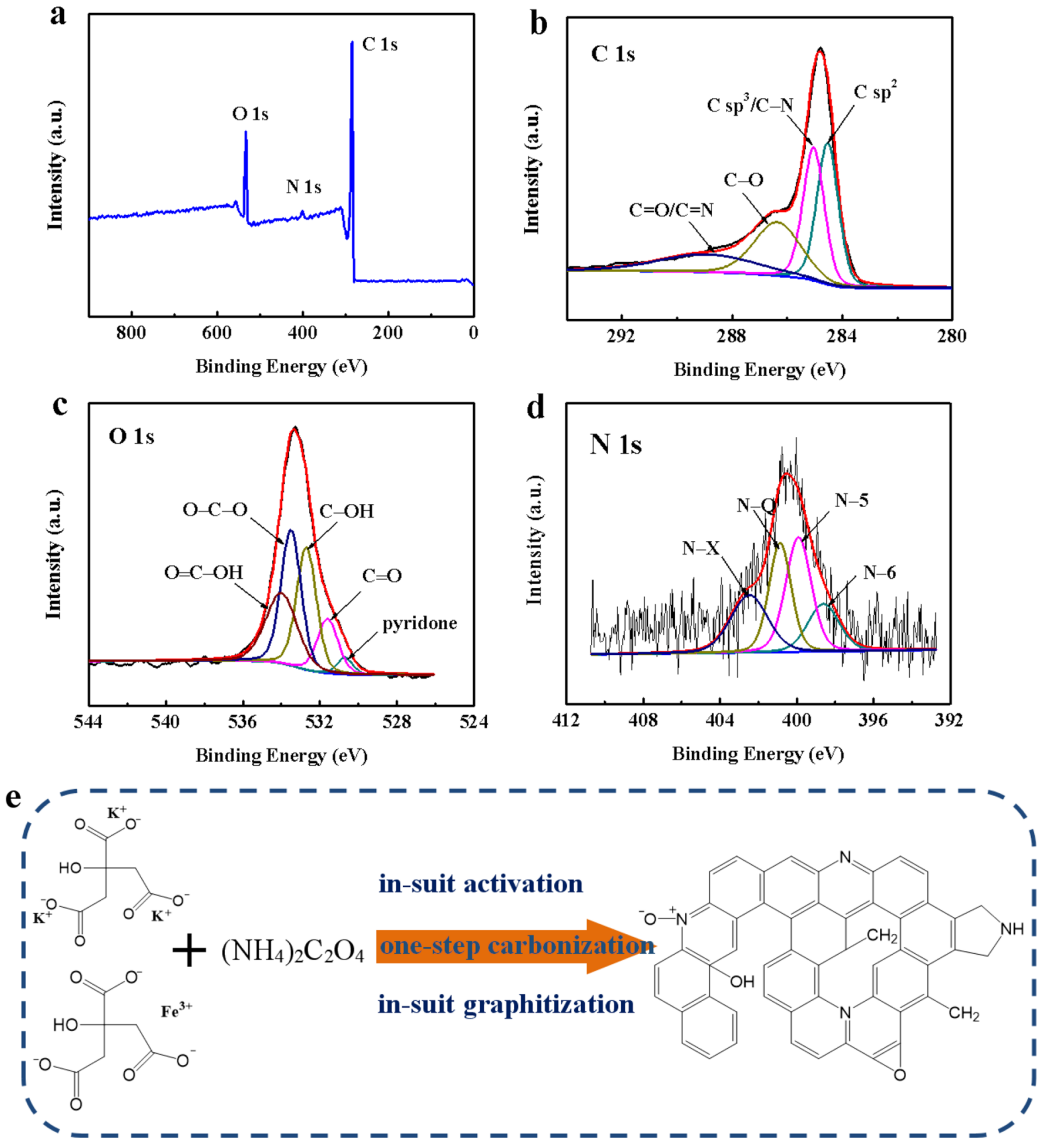
(**a**) XPS survey of HPGC-750; high resolution XPS spectra of (**b**) C 1 s, (**c**) O 1 s, and (**d**) N 1 s for HPGC-750; (**e**) synthetic route to the fabrication of HPGC-*T* by direct pyrolysis potassium citrate and iron citrate. The synthetic route was drawn using Powerpoint 2019 (https://www.microsoft.com/zh-cn/microsoft-365/get-started-with-office-2019).

The surface functional groups of carbon materials were investigated by FTIR spectroscopy (Figure S1). All PC-750, PGC-750, and HPGC-750 have strong and broad bands at ~ 3435 cm^–1^ corresponding to the O–H stretching. The bands at ~ 2916 and ~ 1639 cm^–1^ are ascribed to C–H and C=O functionalities, respectively. The band at 1389 cm^–1^ is attributed to either C–H bending vibration or O–H deformation. The bands at 1123 cm^–1^ can be ascribed to the C–O–C stretching vibration^25^. The XPS survey spectra of HPGC-750 are shown in Fig. 5. In Fig. 5a, two pronounced peaks locate at ~ 284.8 and ~ 533.3 eV, attributed to carbon (C 1 s, 85.10 at%) and oxygen (O 1 s, 11.65 at%), respectively. And a weak peak appeared at ~ 401.7 eV should be attributed to N 1 s, and the N content is 3.24 at%, indicating that NH_3_ gas generated by pyrolysis process of (NH_4_)_2_C_2_O_4_ subsequently doped into HPGC-750 to bring N into carbon matrix. The high-resolution of C 1 s spectra can be deconvoluted into four peaks (Fig. 5b), representing *sp*^2^-bonded carbon (284.5 eV), *sp*^3^-bonded carbon/C–N (285.0 eV), C–O (286.4 eV), and C = O/C = N (288.9 eV). The deconvoluted high-resolution O 1 s XPS spectra (Fig. 5c) exhibit five peaks centered at 530.7, 531.6, 532.7, 533.5, and 534.0 eV, corresponding to pyridone, C=O, C–OH, O–C–O, and O=C–OH, respectively. Four component peaks of nitrogen are found in the XPS spectra of N 1 s (Fig. 5d), indicating four different chemical states of nitrogen atoms in carbon network, including pyridinic–N (398.6 eV), pyrrolic–N (399.9 eV), graphitic–N (400.87 eV), and quaternary–N^+^–O^−^ (402.47 eV)^22,26^, as described in Fig. 5e. Among them, pyridinic–N and pyrrolic–N are electroactive and contribute to providing pseudocapacitance, and therefore enhanced capacitance, whereas graphitic–N can facilitate the electron transfer, improving the conductivity of carbon materials^27^.

### Electrochemical measurements

**Figure 6:**
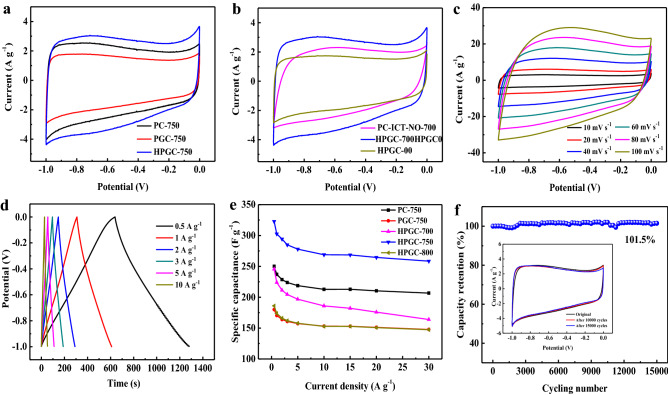
Electrochemical characteristics of a three-electrode system in 6 M KOH: (**a**) CV curves of PC-750, PGC-750, and HPGC-750 at 10 mV s^−1^; (**b**) CV curves of HPGC-*T* at 10 mV s^−1^; (**c**) CV curves of HPGC-750 at various scan rates from 10 to 100 mV s^−1^; (**d**) GCD curves of HPGC-750 under various current densities; (**e**) specific capacitances of all electrodes as a function of current density; (**f**) cycling stability of HPGC-750 for 15,000 cycles measured at 100 mV s^−1^ with inset showing CV curves at 10 mV s^−1^.

Due to honeycomb-like hierarchical porous structure, large accessible surface area, and sufficient heteroatoms, HPGC-750 is expected to be an ideal electrode for supercapacitors. Figure 6a compares the CV curves of PC-750, PGC-750, and HPGC-750 at a current density of 10 mV s^−1^. In the case of HPGC-750, the CV curve displays the largest CV curve area, indicating the highest specific capacitance, evidencing the advantages of 3D honeycomb-like hierarchical porous graphitic carbon. A slight hump is also observed, which is corresponding to the redox reactions of N and O functional groups^21,27^. A pair of sharp peaks at the edge of − 1 and 0 V could be ascribed to limited ion transport and adsorption in some irregular micropores with narrow bottlenecks^28^. HPGC-750 also has the maximum CV curve area among HPGC-*T* samples (Fig. 6b). Although HPGC-700 possesses the largest *S*_BET_, the relatively poor graphitization degree limits electron transfer and charge storage. Figure 6c shows the CV curves of HPGC-750 at various scan rates from 10 to 100 mV s^−1^. With increasing scan rate, a slight deviation from the rectangular nature of CV plots is associated with limited ions diffusion at higher scan rate.

The GCD curves of HPGC-750 are shown in Fig. 6d. The approximately symmetric triangle shapes of curves at all current densities indicate a predominantly double-layer charge storage mechanism. Figure 6e displays the specific capacitances of PC-750, GC-750, and HPGC-*T* at different current densities. HPGC-750 has the highest capacitance of 322.6 F g^−1^ at 0.5 A g^−1^. Besides, it maintains a high capacitance of 258.4 F g^−1^ at an ultrahigh current density of 30 A g^−1^. The high capacitance of HPGC-750 could be attributed to the multiple synergistic effects: (1) hierarchical porous structure integrates the advantages of different pore structures, simultaneously providing ion-buffering reservoirs, rapid ion transfer channels, and abundant locations for ions accumulation; (2) owing to the effects of chemical activation and physical activation, HPGC-750 has a ultrahigh *S*_BET_, providing enough active sides for energy storage; (3) the coexistence of graphitic and amorphous carbon structure could enhance electrical conductivity and improve wettability, benefitting for capacitance improvement. The rate performance is a key parameter to evaluate the electrochemical performance of electrode. In hierarchical porous graphitic texture, micropores provide active sides for charge storage, macropores serve as ion-buffering reservoirs, mesopores act as ion-highways to facilitate fast ion transmission, and graphitic structure provides conductive network for rapid charge transfer. When the current density increased by 30 times, HPGC-750 and HPGC-800 achieve excellent rate capabilities of 80.1% and 79.2%, respectively. The outstanding rate capability can be attributed to large mesopore contribution with bigger pore size and higher graphitization degree those facilitate ion and electron transport especially at high current density. HPGC-700 possesses relatively smaller pore size of mesopores and lower graphitization degree, and exhibits a poor rate capability of 66.7%.

Figure 6f shows the cycling stability of HPGC-750 at a scan rate of 100 mV s^−1^ for 15,000 cycles. In the initial 1750 cycles, the capacitance maintains at 99.2% of the initial capacitance, and then increases to 101.3% after 2500 cycles. The cycling-induced increase of capacitance is also reported in many references, which attributed to the penetration of electrolyte ions and in-situ activation of electrode so as to provide additional active surface area for charge storage^29–31^. After 2500 cycles, the electrochemical stability remains somewhat steady, and it maintains 101.5% of initial capacitance after 15,000 cycles. In comparison with the first CV curve at a scan rate of 10 mV s^−1^, CV curves after 10,000 cycles and after 15,000 cycles still maintain similar shape without obvious distortion, which demonstrates that the electrode has an excellent cycling performance.

Based on the above analysis, 750 °C is an optimum temperature from the view of the morphologies, structures, and electrochemical performances of the carbon materials. On the basis of these, we further investigated the effects of the content of (NH_4_)_2_C_2_O_4_ and the mass ratio of citrate/iron citrate on the structures and capacitive properties of the samples, denoted as HPGC_*x–y–z*_ (where *x, y, z* refer to the mass of citrate, iron citrate, and (NH_4_)_2_C_2_O_4_, respectively). Figure S2 displays the morphologies and structures of HPGC_*x–y–z*_. HPGC_3*–*1.5–6_ shows little macropores (Figure S2a), and HPGC_3*–*1.5–10_ (Figure S2b) maintains 3D honeycomb framework. As for HPGC_2–2.5–8_ (Figure S2c) and HPGC_3.5–1–8_ (Figure S2d), the honeycomb structure is disappeared. With different mass ratio of potassium citrate/iron citrate/(NH_4_)_2_C_2_O_4_, HPGC_*x*–*y–z*_ exhibits tunable *S*_BET_ and pore size distribution (Figure S3, Table S1). The pore sizes of all HPGC_*x*–*y–z*_ are mainly around 1.27 and 2.51 nm. The *S*_BET_ of HPGC_3–1.5–*z*_ decreases with increased content of (NH_4_)_2_C_2_O_4_, while excessive (NH_4_)_2_C_2_O_4_ results in an increased *S*_BET_. HPGC_2–2.5–8_ has a lower *S*_BET_ (1911 m^2^ g^−1^) than that of HPGC_3–1.5–8_ due to insufficient chemical activation. But when the mass ratio of potassium citrate/iron citrate increased to 3.5:1, the *S*_BET_ of HPGC_3.5–1–8_ (2125.7 m^2^ g^−1^) decreases, probably caused by the excessive activation effect of potassium citrate. Figure S4 displays the electrochemical performances of all HPGC_*x*–*y–z*_ electrodes. When the mass ratio of potassium citrate/iron citrate/(NH_4_)_2_C_2_O_4_ is 3:1.5:8, the carbon electrode possesses optimal capacitive performances, although its *S*_BET_ is not largest, indicating that the *S*_BET_ is not completely directly proportional to capacitance.

**Figure 7:**
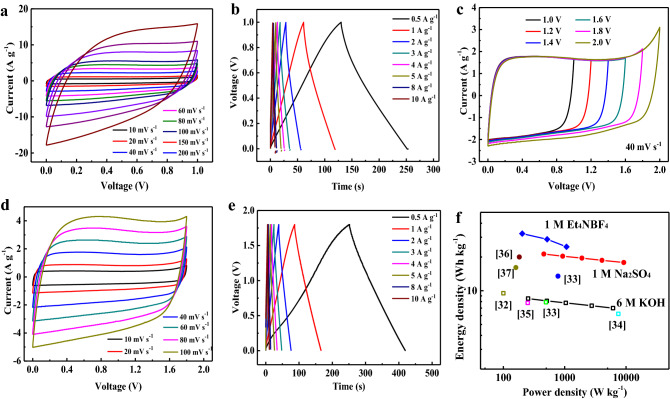
Electrochemical characteristics of HPGC-750 symmetric supercapacitor: (**a**) CV curves at various scan rates in 6 M KOH; (**b**) GCD curves at various current densities in 6 M KOH; (**c**) CV curves tested in different potential windows at 40 mV s^−1^ in 1 M Na_2_SO_4_; (**d**) CV curves at different scan rates in a potential window of 1.8 V in 1 M Na_2_SO_4_; (**e**) GCD curves at different current densities in 1 M Na_2_SO_4_; (**f**) Ragone plots of HPGC-750//HPGC-750 supercapacitor and performances comparison with other carbon-based symmetric supercapacitors (circle: 6 M KOH electrolyte, and sphere: 1 M Na_2_SO_4_ electrolyte).

With 3D hierarchical porous texture and localized graphitic structure, CV curves of symmetric supercapacitor HPGC-750//HPGC-750 performed in 6 M KOH show rectangular shapes at various scan rates (Fig. 7a). The rectangular nature even at a higher scan rate of 200 mV s^−1^ is related to the presence of mesopores. All the GCD curves in Fig. 7b show regular symmetric triangle, which again confirms the double-layer formation at the electrode–electrolyte interface. The maximum specific capacitance for a single electrode calculated from GCD curves is 245.1 F g^−1^ at 0.5 A g^−1^ and it retains 201.6 F g^−1^ at 10 A g^−1^, corresponding to 82.2% capacitance retention.

The symmetric supercapacitor HPGC-750//HPGC-750 was also tested in 1 M Na_2_SO_4_, owing to its lower H^+^ and OH^−^, which allows it providing a larger operating voltage. Figure 7c shows the CV curves of HPGC-750//HPGC-750 at 40 mV s^−1^ within different potential windows, and they present well reversible cycle even at a potential window of 0−1.8 V. The rectangle-like CV curves (Fig. 7d) and symmetrical GCD curves (Fig. 7e) within the voltage windows 0−1.8 V further demonstrate excellent rate capability. In 1 M Et_4_NBF_4_/AN, the rectangular nature of CV curves (Figure S5a) and the linear and symmetrical characteristics of GCD curves at various current densities (Figure S5b) further reveal the electrical double-layer capacitance behavior. The Ragone plots in Fig. 7f show that symmetric supercapacitor HPGC-750//HPGC-750 has an excellent energy density of 21.25 W h kg^−1^ at 456.8 W kg^−1^ in 1 M Na_2_SO_4_ electrolyte, and it still retains 14.4 W h kg^−1^ at a high power density of 39,875.6 W kg^−1^. In 6 M KOH electrolyte, the energy density is 8.5 W h kg^−1^ at a power density of 253.0 W kg^−1^. Due to large potential window, the supercapacitor can achieve a high energy density of 32.3 W h kg^−1^ at a power density of 203.0 W kg^−1^ in 1 M Et_4_NBF_4_/AN. The energy and power values are comparable or even superior to those of the previously reported carbon materials^32–37^. This is attributed to the honeycomb-like graphitic carbon skeleton benefiting the mass transport and electron transfer through the open pore channels and interconnected carbon network.

**Figure 8:**
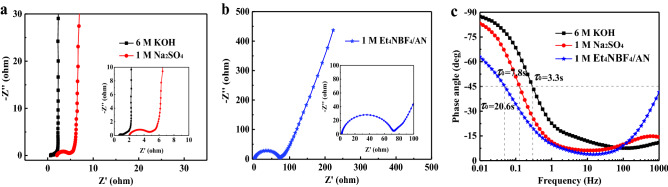
(**a**) Nyquist plots of HPGC-750 symmetric supercapacitors in 6 M KOH and 1 M Na_2_SO_4_, (**b**) Nyquist plots of HPGC-750 symmetric supercapacitors in 1 M Et_4_NBF_4_/AN, and (**c**) Bode phase angle plots of HPGC-750 symmetric supercapacitors in different electrolytes.

Electrochemical impedance spectroscopy was also measured to evaluate the ion and electron transport kinetics in different electrolytes. Figure 8a shows the Nyquist plots of HPGC-750 supercapacitors in 6 M KOH and 1 M Na_2_SO_4_ and Fig. 8b shows the Nyquist plots in 1 M Et_4_NBF_4_/AN. The vertical nature of curves in the low frequency region indicates nearly ideal capacitive performance. The semicircle in high frequency region is related to charge transfer resistance (*R*_ct_). It is observed that the diameters of the semicircles for aqueous electrolytes are much smaller than that for organic electrolyte. The value of equivalent series resistance (*R*_s_) for KOH electrolyte is calculated to be 0.60 Ω, and it is 2.07 Ω for 1 M Na_2_SO_4_, 2.14 Ω for 1 M Et_4_NBF_4_/AN. The smaller *R*_s_ and *R*_ct_ for aqueous electrolytes than that for organic electrolyte could be ascribed to high conductivity, small ion size, and quick ionic mobility of aqueous electrolyte^38^. Figure 8c shows the Bode phase angle plots. The large phase angles (− 87.2° for KOH and − 82.5° for Na_2_SO_4_) at low frequency indicate mainly electrical double-layer capacitance. The relaxation time constants (*τ*_0_ = 1/*f*, at the phase angle of − 45°) are 7.8 s for KOH and 3.3 s for Na_2_SO_4_ electrolyte. The fast frequency response indicates the fast charge–discharge rate. However, for 1 M Et_4_NBF_4_/AN electrolyte, the relaxation time constant is 20.6 s because of larger size of ion and higher electrolyte resistance of organic electrolyte.

## Conclusion

In summary, hierarchical porous graphitic carbon with interconnected framework has been successfully synthesized by in situ activation–graphitization method through only one-step pyrolysis of potassium citrate, ferric citrate, and (NH_4_)_2_C_2_O_4_. The obtained HPGC-750 balances the relationship between pore structure and electrical conductivity. It possesses favorable characteristics, such as high *S*_BET_ of 2973.3 m^2^ g^−1^, good conductivity, and hierarchical porous structure facilitating ion transportation and accommodation in electrodes. Such unique architecture endows HPGC-750 with excellent electrochemical performances: a high capacitance of 322.6 F g^−1^ at 0.5 A g^−1^, good rate capability with 258.4 F g^−1^ at 30 A g^−1^, and an outstanding cycle stability (no loss after 15,000 cycles). Supercapacitor prepared with HPGC-750 shows a high energy density 21.3 W h kg^−1^ at a high power density of 456.7 W kg^−1^ in 1 M Na_2_SO_4_ electrolyte. This study provides a promising strategy to develop hierarchical porous graphitic carbon for high-performance of supercapacitors and other energy researches such as fuel cell, catalysis, and so on.

## Methods

### Materials

Iron citrate (FeC_6_H_5_O_7_·5H_2_O) was provided by Aladdin Industrial Co., Ltd. Potassium citrate tribasic monohydrate (C_6_H_5_K_3_O_7_·H_2_O) and diammonium oxalate monohydrate [(NH_4_)_2_C_2_O_4_·H_2_O] were purchased from Tianjin Tianli Chemical Reagent Co., Ltd. Potassium hydroxide (KOH), sodium sulfate (Na_2_SO_4_) and hydrochloric acid (HCl) were provided by Tianjin Fengchuan Chemical Reagent Technologies Co. Ltd. All the chemical reagents were used as received. Deionized water was used throughout the experiments.

### Preparation of materials

In a typical synthesis, 3 g potassium citrate tribasic monohydrate, 1.5 g iron citrate, and 8 g diammonium oxalate monohydrate were grinded homogeneously to achieve a sufficient contact. Subsequently, the mixture was carbonized in a N_2_ atmosphere at high temperatures (700, 750, and 800 °C) for 2 h at a heating rate of 5 °C min^−1^. The calcined products were treated with 3 M HCl solution and then washed with deionized water several times. After dried at 80 °C, the final product HPGC-*T* was obtained (where *T* represents carbonization temperature).

For comparison, the sample synthesized by solely pyrolysis potassium citrate at 750 °C was denoted as PC-750. To evaluate the effect of (NH_4_)_2_C_2_O_4_ on the structure of the resultant carbon materials, synthesis without (NH_4_)_2_C_2_O_4_ was also performed, and the resultant sample was labeled as PGC-750.

### Materials characterization

Scanning electron microscopy (SEM, JSM-6510F) was conducted to investigate and morphologies and structures of the samples. Transmission electron microscopy (TEM) was taken using FEI-G2F20. X-ray diffraction (XRD) patterns were obtained by Miniflex 600 diffractometer. Raman spectra were measured by using a Jobin–Yvon, HR 800 spectrometer. The N_2_ adsorption–desorption isotherms were obtained by using Micromeritics ASAP 2460 instrument^39^. Surface chemical composition of the sample was investigated by X-ray photoelectron spectroscopy (XPS, ESCALAB 250) and fourier transformation infrared spectroscopy (FTIR, Bruker Tensor 27).

### Electrochemical measurement

The electrochemical performances of samples were evaluated by cyclic voltammetry (CV), galvanostatic charge–discharge (GCD) and electrochemical impedance spectroscopy (EIS) through a CHI760e electrochemical workstation. Active carbon material (2.4 mg, 80 wt%), acetylene black (10 wt%), and polytetrafluoroethylene (10 wt%) were mixed and coated on a nickel foam to prepare a working electrode. In a three-electrode system, Hg/HgO and Pt foil electrodes were used as the reference electrode and the counter electrode, respectively. The electrolyte was 6 M KOH solution. The specific capacitance *C* (F g^–1^) in the three-electrode system can be obtained by following equation:

1 $$ C = I \cdot \Delta t/\left( {m \cdot \Delta V} \right) $$

where *I*, Δ*t*, *m*, and Δ*V* are the discharge current (A), the discharge time (s), the mass of active material (g), and the potential window (V), respectively.

In two-electrode system, the cell consists of two symmetric electrodes with equal mass were separated by a polypropylene membrane using 6 M KOH, 1 M Na_2_SO_4_, or 1 M Et_4_NBF_4_/AN electrolyte. The specific capacitance *C*_sp_ (F g^–1^) for a single electrode, energy density *E* (W h Kg^–1^), and power density *P* (W Kg^–1^) of supercapacitor were calculated according to the following Eqs. (2)–(4):

2 $$ C_{{{\text{sp}}}} = { 4}I \cdot \Delta t/\left( {m \cdot \Delta V} \right) $$

3 $$ E = C_{{{\text{sp}}}} \cdot V^{{2}} /{8} $$

4 $$ P \, = \, E/\Delta t $$

where *m* is the total mass of active materials for two electrodes (g), *V* is the maximum discharging potential (V)^39^.

## Supplementary Information

Supplementary Information
