# Supplementary material for: In situ activation graphitization to fabricate hierarchical porous graphitic carbon for supercapacitor
Source: Sci Rep. 2021 Mar 25;11:6825. doi: 10.1038/s41598-021-85661-0 (PMC7994305; doi:10.1038/s41598-021-85661-0)
Supplement: Supplementary file 1 — Supplementary Information [file 41598_2021_85661_MOESM1_ESM.doc]

**Supplementary Information**

***In situ* activation graphitization to fabricate** **hierarchical porous graphitic carbon for supercapacitor**

Yanling Zhao ^1*^and Xiaohua Zhang ^2*^

^1^ Department of Medical Imaging, Shanxi Medical University, Taiyuan 030001, China.^2^ College of Materials Science and Engineering, Taiyuan University of Science and Technology, Taiyuan 030024, China. Correspondence and requests for materials should be addressed to Y. Z. (email: yxzyl1998@126.com) or X. Z. (email: xiaohuaz08@163.com).

Figure S1 FTIR spectra of PC-750, PGC-750, and HPGC-750


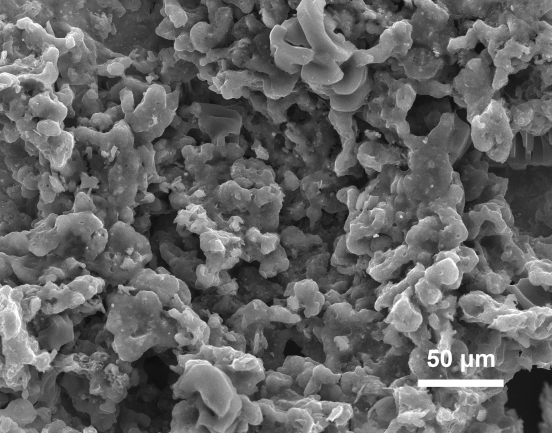


**a**


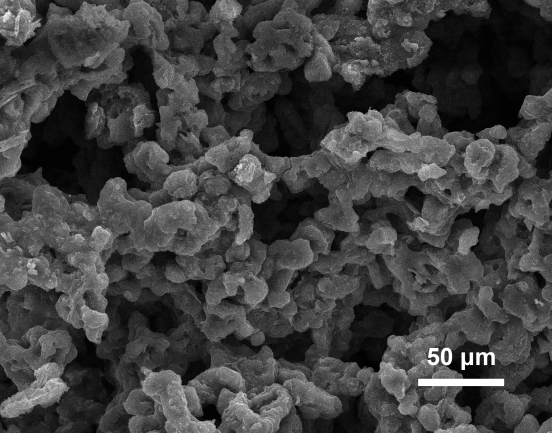


**b**


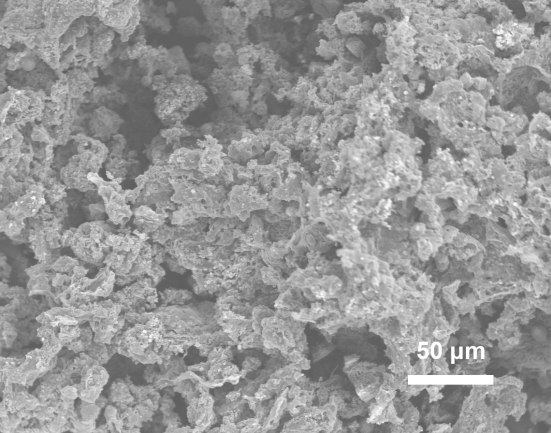


**c**


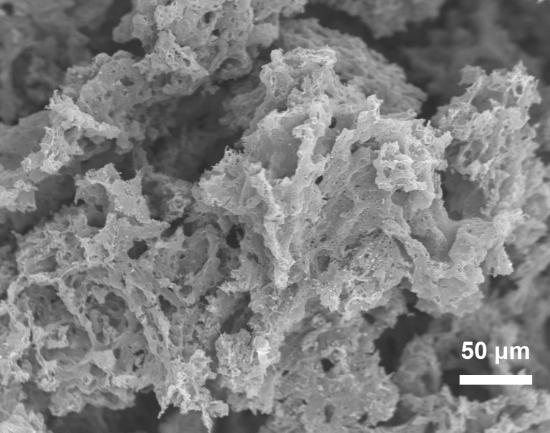


**d**

Figure S2 SEM images of (a) HPGC_3-1.5-6_, (b) HPGC_3-1.5-10_, (c) HPGC_2-2.5-8_, and (d) HPGC_3.5-1-8_. Photographs compiled using Powerpoint 2019 (https://www.microsoft.com/zh-cn/microsoft-365/get-started-with-office-2019) without changing the content of images themselves.

Figure S3 (a) N_2_ adsorption-desorption isotherms and (c) the pore size distribution curves of HPGC_3-1.5-6_, HPGC_3-1.5-8_, and HPGC_3-1.5-10_; (b) N_2_ adsorption-desorption isotherms and (d) the pore size distribution curves of HPGC_2-2.5-8_, HPGC_3.5-1-8_, and HPGC_3-1.5-8_*.*

Table S1 Porosity properties of as-prepared HPGC_x-y-z_ samples.

| Samples | *S*_BET_  (m^2^ g^−1^) | *S*_mic_^a^  (m^2^ g^−1^) | *S*_mes_^b^  (m^2^ g^−1^) | *V*_mic_^c^  (cm^3^ g^−1^) | *V*_total_^d^  (cm^3^ g^−1^) | *D*_ap_^e^  (nm) |
| --- | --- | --- | --- | --- | --- | --- |
| HPGC_3-1.5-6_ | 3129.2 | 1716.5 | 1412.7 | 1.64 | 1.80 | 2.30 |
| HPGC_3-1.5-10_ | 3311.9 | 1785.7 | 1526.2 | 1.65 | 1.82 | 2.20 |
| HPGC_2-2.5-8_ | 1911.7 | 1027.3 | 884.4 | 0.98 | 1.13 | 2.36 |
| HPGC_3.5-1-8_ | 2125.7 | 1154.7 | 971 | 1.13 | 1.29 | 2.42 |

Note: ^a^ micropore surface area; ^b^ mesopore surface area; ^c^ micropore volume; ^d^ total pore volume; ^e^ average pore size.

Figure S4 Electrochemical characteristics of HPGC*_x-y-z_* electrodes in a three-electrode system using 6 M KOH electrolyte: (a) CV curves of HPGC_3-1.5-6_, HPGC_3-1.5-8_, and HPGC_3-1.5-10_ at 10 mV s^−1^; (b) CV curves of HPGC_2-2.5-8_, HPGC_3-1.5-8_, and HPGC_3.5-1-8_ at 10 mV s^−1^; (c) GCD curves of HPGC*_x-y-z_* under various current densities; (d) specific capacitances of all HPGC*_x-y-z_* electrodes as a function of current density.

Figure S5 Electrochemical characteristics of HPGC-750 symmetric supercapacitor in 1 M Et_4_NBF_4_/AN: (a) CV curves at various scan rates; (b) GCD curves at various current densities.
